# The intersection of Evaluation and Geriatric Education

**DOI:** 10.1093/geroni/igaf122.1372

**Published:** 2025-12-31

**Authors:** Rhiannon Williams, Christina Cauble, Miranda Moore

## Abstract

The intersection of geriatric education and evaluation plays a critical role in shaping high-quality geriatric learning environments, ensuring that educational programs effectively prepare learners to advance the quality of care for older adults while strategically communicating their value to key stakeholders who can drive investment and prioritization. The number of older adults in the United States continues to grow, while many healthcare professionals are inadequately trained to provide care to this population. Through the use of cases, this session explores how evaluative thinking drives design, implementation, and outcomes of geriatric education programs. Unfortunately, evaluation is often just used to determine outcomes. Evaluative thinking and the use of a logic model provides a critical lens and framework for program development. First, we provide a brief overview of what a logic model is and how it can be used from inception of a learning program to the end. Next, we focus on a rubric tool development and use for geriatric education products/ outputs such as written work, presentations, or other projects. Then, we examine the assessment of short-term student learning outcomes, using a reflective tool as a means of both facilitating student learning as well as assessing their learning. Finally, we address how to create and share evaluation output or outcome data in a visual format that diverse stakeholders can utilize to ensure transparency, accountability, and collaboration. Attendees will leave with actionable insights on how to systematically evaluate educational efforts and drive meaningful improvements in geriatric education.

